# A Large Web-Based Observer Reliability Study of Early Ischaemic Signs on Computed Tomography. The Acute Cerebral CT Evaluation of Stroke Study (ACCESS)

**DOI:** 10.1371/journal.pone.0015757

**Published:** 2010-12-30

**Authors:** Joanna M. Wardlaw, Rüdiger von Kummer, Andrew J. Farrall, Francesca M. Chappell, Michael Hill, David Perry

**Affiliations:** 1 SINAPSE Collaboration, Division of Clinical Neurosciences, University of Edinburgh, Edinburgh, United Kingdom; 2 Department of Neuroradiology, University Hospital, Technische Universität Dresden, Dresden, Germany; 3 Stroke Unit, Department of Neurosciences, University of Calgary, Foothills Medical Centre, Calgary, Canada; Julius-Maximilians-Universität Würzburg, Germany

## Abstract

**Background:**

Early signs of ischaemic stroke on computerised tomography (CT) scanning are subtle but CT is the most widely available diagnostic test for stroke. Scoring methods that code for the extent of brain ischaemia may improve stroke diagnosis and quantification of the impact of ischaemia.

**Methodology and Principal Findings:**

We showed CT scans from patients with acute ischaemic stroke (n = 32, with different patient characteristics and ischaemia signs) to doctors in stroke-related specialties world-wide over the web. CT scans were shown twice, randomly and blindly. Observers entered their scan readings, including early ischaemic signs by three scoring methods, into the web database. We compared observers' scorings to a reference standard neuroradiologist using area under receiver operator characteristic curve (AUC) analysis, Cronbach's alpha and logistic regression to determine the effect of scales, patient, scan and observer variables on detection of early ischaemic changes. Amongst 258 readers representing 33 nationalities and six specialties, the AUCs comparing readers with the reference standard detection of ischaemic signs were similar for all scales and both occasions. Being a neuroradiologist, slower scan reading, more pronounced ischaemic signs and later time to CT all improved detection of early ischaemic signs and agreement on the rating scales. Scan quality, stroke severity and number of years of training did not affect agreement.

**Conclusions:**

Large-scale observer reliability studies are possible using web-based tools and inform routine practice. Slower scan reading and use of CT infarct rating scales improve detection of acute ischaemic signs and should be encouraged to improve stroke diagnosis.

## Introduction

Computerised tomography (CT) brain scanning is widely available.[1], [2] It is quick and can be used in virtually all patients so is the main brain imaging method in patients with acute stroke. However, the plain CT brain scan is not well appreciated in stroke because early CT changes associated with brain ischaemia are subtle.

Arterial occlusion leading to ischaemic brain tissue damage is associated with a net uptake of water (ionic oedema),[3], [4] that can be detected with CT as tissue hypoattenuation.[5] In acute ischaemic stroke, ionic oedema may be present or not depending on the degree and duration of ischaemia. Consequently, hypoattenuation of ischaemic gray matter on CT, representing the increase in tissue water content, may be present or not. CT is highly sensitive and specific for changes in tissue water content and thus ischaemic damage.[6] Another CT finding in acute ischaemic stroke is brain tissue swelling without a change in x-ray attenuation, representing an area of low perfusion pressure and compensatory vasodilation prior to development of ionic oedema.[7] Additionally, the occluded artery may be hyperattenuated representing thrombo-embolism.[8]

Within the first six hours of stroke onset, the detection of tissue hypoattenuation and/or swelling and hyperattenuated arteries requires training, experience and understanding of the underlying pathology. These signs on CT are generally regarded as being difficult to detect. Inter-observer agreement for “any early CT ischaemic sign” was between 0.14 to 0.78 (kappa); sensitivities and specificities for ischaemic sign detection was 20 to 87% and 56 to 100% respectively.[9], [10]

Several scales have been developed to classify visible ischaemic changes on CT scans,[11]–[14] which may also improve detection of ischaemic signs, indicate prognosis and guide treatment. However, these have generally not been tested in large scale studies with multiple observers designed to reflect routine practice. Having large numbers of observers of different backgrounds read large numbers of scans is a logistic challenge, only achievable under exceptional circumstances.[11] Previous studies of observer reliability for acute infarct detection on CT had a median of five observers and 30 scans, so had inadequate power to examine the effect of important observer, scan, or patient characteristics that might influence lesion detection.[9] Although some rating scales for early ischaemic changes have been tested in individual studies, there has been no comparison of the most commonly used rating scales.

We established a large observer reliability study using web technology to improve understanding of observer detection of CT ischaemic signs, the Acute Cerebral CT Evaluation of Stroke Study (ACCESS).[10] We previously reported which early CT ischaemic signs (hypoattenuation, swelling or hyperattenuated artery) were best detected and performed a simple analysis of observer characteristics by comparing neuroradiologists to other specialties.[10] We now determine, with more observers, and a more complex statistical approach, whether use of any ischaemic stroke lesion classification scores improves observer detection of CT signs of ischaemia, and which, if any, patient-related, stroke-related or scan-related factors affect ischaemic lesion detection. Better detection of signs associated with ischaemia on CT would improve doctors' confidence in the early diagnosis of ischaemic stroke and its specific pathology, and might improve use of thrombolytic treatment.

## Methods

The study was conducted using Standards for Reporting of Diagnostic Accuracy (STARD) principles.[15] We established an internet-based scan reading tool (www.neuroimage.co.uk) to maximise the number and range of observers and scans as described previously.[10] Brief details of key study methods are given here.

### CT scans

We selected CT scans stratified for patient characteristics (age, duration of symptoms, stroke severity) and specific signs. We randomly chose 22 scans representing: time to scanning (half <3 hours; half 3–6 hours after stroke); patient age (half <70; half >70 years); and stroke severity (National Institutes of Health Stroke Scale Score, NIHSS: half ≤12; half >12), independently of which signs the scans showed, from 120 stroke patients admitted sequentially to a teaching hospital and scanned within six hours of acute stroke. We also chose scans showing specific early ischemic changes (n = 10), independent of patient characteristics, from previous[16] and ongoing[17] trials of thrombolysis in stroke. The scans were anonymised and stored electronically in Digital Jacket™ (DesAcc, Inc, 801 W Adams St, Chicago IL 60607, USA) in Joint Photographic Experts Group (JPEG) format, optimised for gray/white matter differentiation on CT, for streaming over the web during scan reading. All 32 scans except one were shown twice, in random order, without informing the observers of duplicate scan viewing, making a total of 63 scan assessments.

### Ethical approval

The three primary studies from which the CT scans were chosen were approved by their respective Ethics Committees (The Ethics Committee, University Hospital, Mannheim, Germany; the Ethics of Medical Research Committee, Southern General Hospital Glasgow; the Scotland Multicentre Research Ethics Committee A), including use of anonymised scans in secondary relevant analyses. All patients gave written informed consent or, in the case of patients who were not able through the effects of the acute stroke to give consent themselves, written assent was obtained from their relative, as approved by the respective ethics committees.

### Observers

We sought as many observers as possible through stroke, neurology, neuroradiology and other relevant conferences, newsletters of trials and professional organizations and journal articles. We encouraged participation by awarding 5 Continuing Professional Development (CPD) credits from the UK Royal Colleges for reading all scans, and several monetary prizes for the fastest readers.

### Scan reading

Observers registered their specialty, years of training in that specialty and country of origin, optimised monitor settings (contrast/brightness) and ambient light for detecting subtle grey scale differences with an “SMPTE” test (Society of Motion Picture Television Engineers), on the study website. They read a test scan to familiarise themselves with the web scan viewing tool. Thereafter, batches of scans were assigned and observers read the scans blind to patient and clinical stroke features. The time taken to complete the questionnaire was recorded. The reading session timed out after five minutes to avoid excessively long apparent reading times through observers being interrupted during a reading.

### Structured questionnaire

The questionnaire was developed and tested on acute stroke CT scans outside the study cohort. The signs of early ischaemia on CT are: a) decreased parenchymal x-ray attenuation, b) tissue swelling (mass effect), and c) hyperattenuated artery sign (due to acute arterial occlusion with thrombus).[18] The final questionnaire recorded scan quality, any change in attenuation or swelling (and whether mild or severe), the arterial territory(s) affected, three scoring systems (1/3 middle cerebral artery (MCA) rule,[12] Third International Stroke Trial (IST-3) method,[14] and the Alberta Stroke Program Early CT Score, (ASPECTS)[13]), whether there was any hyperattenuated artery or other abnormality (atrophy, tumour, haemorrhage, old infarct) and the observer's opinion of scan quality (good, moderate, poor) in terms of ease of reading (e.g. straightness of head position, absence of movement artefact). The IST-3 method classifies the ischaemic lesion location (by arterial territory), extent (by typical divisions of the arterial territory, e.g. up to 8 for the MCA) and swelling (ordinal 7-point scale) with diagrams provided for comparison.[14] We defined: “mild hypoattenuation” as grey matter reduced to that of the patient's normal white matter attenuation; “severe hypoattenuation” as grey and white matter attenuation less than the patient's normal white matter; “mild swelling” as effacement of the ipsilateral cortical sulci or slight effacement of the lateral ventricle: and “severe swelling” as complete effacement of the lateral ventricle or midline shift.

### Statistical analysis

We analysed data from readers who had completed all 63 assessments by October 2008. The readings of one neuroradiologist, very experienced in interpretation of CT in acute stroke, were used as the reference standard. We compared each reader's scan readings to the reference standard, by their score on the 1/3 MCA, ASPECTS and IST-3 scales, by calculating the area under receiver operator characteristic (ROC) curves (AUC) and 95% confidence intervals (CI) for the 1^st^ and 2^nd^ assessments. We used Dorfman-Berbaum-Metz Multi-Reader Multi-Case (DBM MRMC) software to calculate and compare the AUCs as this software is designed to calculate AUCs and deal with having multiple observers per scan and multiple scans per observer (http://krl.bsd.uchicago.edu/roc_soft6.htm).[19], [20] The AUC value represents the probability that a patient with the feature in question (e.g. a hyperattenuated artery) will get a more abnormal score than a patient without the feature in question, the presence of the feature in question having been decided by the reference standard. The DBM MRMC method employs jackknifing and analysis of variance (ANOVA) techniques and allows the conclusions drawn from a study to be generalized to both a population of readers and a population of cases. We compared the AUC values for the scales to one another and between 1^st^ and 2^nd^ assessments.

We compared the similarity between scales for the 1^st^ scan readings for detecting ischaemic changes using Cronbach's alpha (α) calculated using Statistical Analysis Software (SAS, www.sas.com) v.9.1. The maximum α is 1; an α of >0.70 indicates that the scales are measuring the same quantity.

We used logistic regression (PROC GENMOD in SAS v.9.1), which accounted for clustering of data both within-reader and within-scan, allowing data from both occasions to be used, to compare the effect of reader, scan and patient characteristics on the probability of agreeing with the reference standard about the detection of any early ischaemic signs without and then when using a scale. The other scales showed very similar patterns for scan characteristics and therefore only the 1/3 MCA was examined in depth as representative of the other two.

## Results

The analysis is based on 258 observers who completed all 63 assessments, representing 33 nationalities and six major specialties. The majority of observers were neurologists (113, 44%), then geriatricians (39, 15%), general radiologists (33, 13%), neuroradiologists (25, 10%), stroke physicians (21, 8%) and others (including emergency physicians, family doctors, 27, 10%). Half the readers had been reading stroke CT scans in clinical practise from between five and 15 years.

Patients had a mean age of 70.3 years (95% CI 65.3 to 75.4) and median NIHSS score of 9.5 (95% CI 6.3 to 12.7). Twenty patients presented with ischaemia in the MCA territory. The median time to scan was 2.3 hours (range 1 to 5.7 hours).

The scales appeared to be measuring the underlying degree of CT ischaemic change consistently and reliably according to Cronbach's α: IST-3 versus 1/3 MCA (α = 0.95) and versus ASPECTS (α = 0.95) were similar to 1/3 MCA versus ASPECTS (α = 0.93).

There was no difference in the performance of the three infarct rating scales when each observer was compared with the reference standard observer using AUC analysis (Table 1). The average AUC for all observers grouped together for the first scan reading by 1/3 MCA (0.602, 95% CI 0.591 to 0.614) was the same as for IST-3 (0.604, 95% CI 0.593 to 0.616) and ASPECTS (0.604, 95% CI 0.592 to 0.616). The figures for the second reading were very similar (Table 1).

**Table 1:** Area under receiver operator characteristic curve (AUC) comparing individual observers with the reference standard for each scoring method.

Scale		AUC	95% CI	Difference time 1–2	p-value
1/3 MCA	1^st^ reading	0.602	0.591,0.614	−0.0024	0.64
	2nd reading	0.604	0.593,0.616		
IST-3	1^st^ reading	0.604	0.593,0.616	0.0045	0.38
	2nd reading	0.600	0.589,0.611		
ASPECTS	1^st^ reading	0.604	0.593,0.616	−0.0038	0.46
	2nd reading	0.601	0.589,0.612		

CI  =  confidence interval; MCA  =  middle cerebral artery; IST-3  =  Third International Stroke Trial (IST-3); ASPECTS  =  Alberta Stroke Program Early CT Score

**Figure 1:**
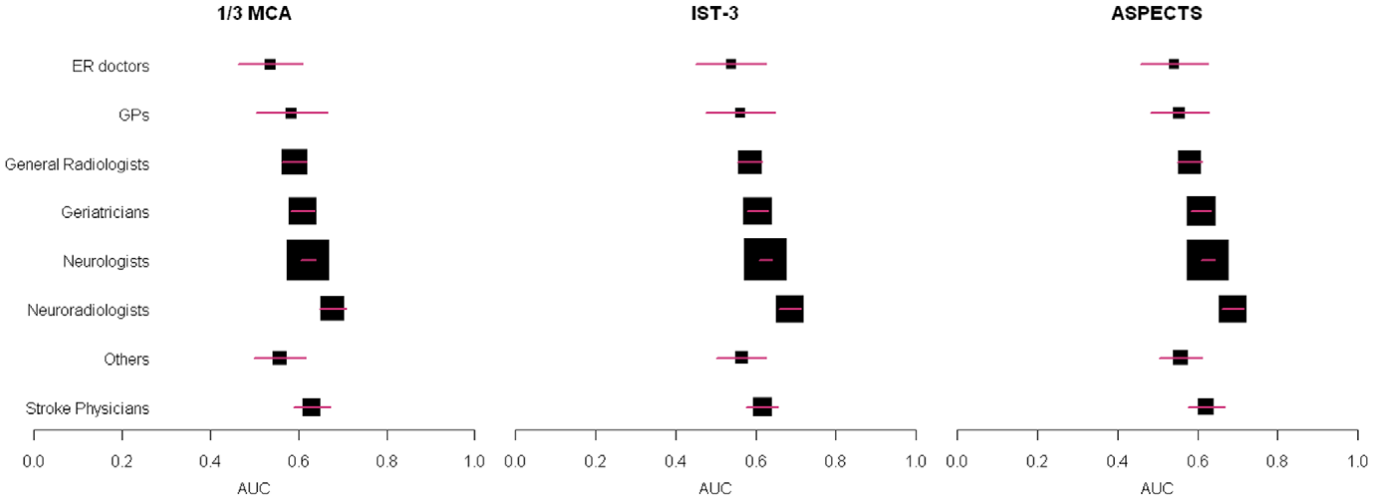
Agreement between observers and the reference standard by observer specialty. A box and whisker plot is shown for each of the 1/3 MCA, IST-3 and ASPECTS scales, with the observer groups listed on the left hand side. Each box and whisker represents the point estimate of the area under the curve (AUC, on x-axis, box) and 95% confidence intervals (whisker) for the observers in that group compared with the reference standard. A larger box indicates that there were more observers in that group. ER doctors  =  emergency doctors; GPs  =  general practitioners or family physicians.

There were differences in the degree of agreement of each specialty with the reference standard reader according to the AUC analysis, but the ordering of agreement by specialty was the same for each infarct rating scale (Figure 1). Thus neuroradiologists had the largest AUC indicating the closest agreement with the reference standard, followed by stroke physicians, neurologists, geriatricians and general radiologists, across all three scales.

There was no suggestion of any learning effect as there was no difference between scales at first and second assessment (p = 0.64, 0.38, 0.46 respectively for 1/3 MCA, IST-3, and ASPECTS). All differences in the AUCs for first and second readings and between scales were negligible (<0.005).

**Figure 2:**
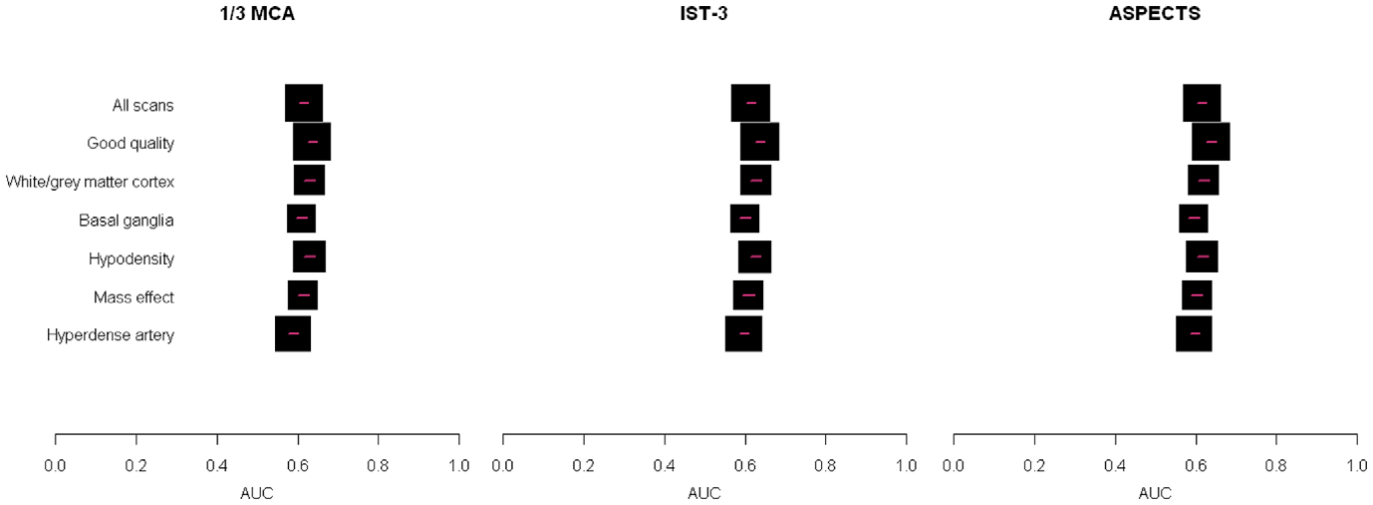
Agreement between observers and the reference standard by scan features. A box and whisker plot is shown for each of the 1/3 MCA, IST-3 and ASPECTS scales, with the scan features listed on the left hand side. Each box and whisker represents the point estimate of the area under the curve (AUC, on x-axis, box) and 95% confidence intervals (whisker) for the observers in that group compared with the reference standard.

We then examined the effect of scan characteristics (presence of ischaemic sign, background appearance of the brain, time to scan), patient (age, NIHSS) and observer characteristics (specialty group, years of training, time to read scan) on each observers' agreement with the reference standard for presence of any acute ischaemic signs and then their agreement on the rating point on each scale (Table 2). All three scales performed very similarly (Figure 2). Due to the layout of the questionnaire there were more answers for the 1/3 MCA scale than for the other two scales, and therefore we only present the results in detail for the 1/3 MCA scale.

**Table 2:** Effect of scan, patient and observer characteristics on agreement between observers and the reference standard.

Agreement on:	Acute Ischaemia		Rating point on 1/3 MCA scale	
	Odds Ratio	p-value	Odds Ratio	p-value
Increasing hypoattenuation (3 point scale)	1.89	<0.0001	0.66	<0.0001
Increasing swelling (5 point scale	1.36	<0.0001	1.56	0.0001
Hyperattenuated artery (yes)	1.47	<0.0001	1.2	<0.0002
Old lesion present	1.33	<0.0001	0.94	0.028
Time to scan	1.13	<0.0001	1.19	<0.0001
Leukoaraiosis	0.59	<0.0001	1.85	<0.0001
Scan quality	1.04	0.3331	0.97	0.5007
Increasing age (effect per year)	1.01	0.0039	1.02	<0.0001
NIHSS	1.00	0.4804	0.99	0.0017
Neuroradiologists versus the rest	1.41	0.0025	1.34	0.0021
Read scan more slowly(per minute)	1.01	<0.0001	1.01	<0.0001
Years in training	0.99	0.1461	0.99	0.2943

An odds ratio of greater than one indicates increasing agreement and of less than one decreasing agreement with the reference standard reader on the presence of acute ischaemia or the rating point on the 1/3 MCA scale (note the pattern was similar for all three scales therefore only 1/3 MCA scale shown).

MCA  =  middle cerebral artery; NIHSS  =  National Institutes of Health Stroke Scale.

Factors which affected detection of acute ischaemic signs, in general, also affected agreement on the 1/3 MCA scale, with few exceptions. Amongst scan characteristics, more severe ischaemic changes (hypoattenuation, swelling, hyperattenuated artery) and increasing time to scanning all increased the odds of agreeing with the reference standard on acute ischaemic change. However, while more swelling, a hyperattenuated artery and increasing time to scanning also increased the odds of agreeing with the reference standard's rating on the 1/3 MCA scale, increasing hypoattenuation decreased the odds of agreeing with the reference standard as to the scale rating. Scan quality (i.e. whether the observer rated the scan as being of good, moderate or poor quality for reading) had no effect. White matter lesions reduced the odds of agreeing with the reference standard on acute ischaemic change but increased the odds of agreeing on the 1/3 MCA scale rating. Old stroke lesions increased the odds of agreeing on acute ischaemia but decreased the odds of agreeing on the scale rating. Amongst patient characteristics, increasing age increased the odds of identifying acute ischaemic change and of agreeing with the reference standard on the scale rating, but stroke severity as assessed by the NIHSS had no effect. Amongst observer characteristics, being a neuroradiologist and longer times to read the scans both increased the odds of agreeing about the presence of acute ischaemic signs with the reference standard and of agreeing with the reference standard's scale rating. However, years of experience of reading scans had no effect.

**Figure 3:**
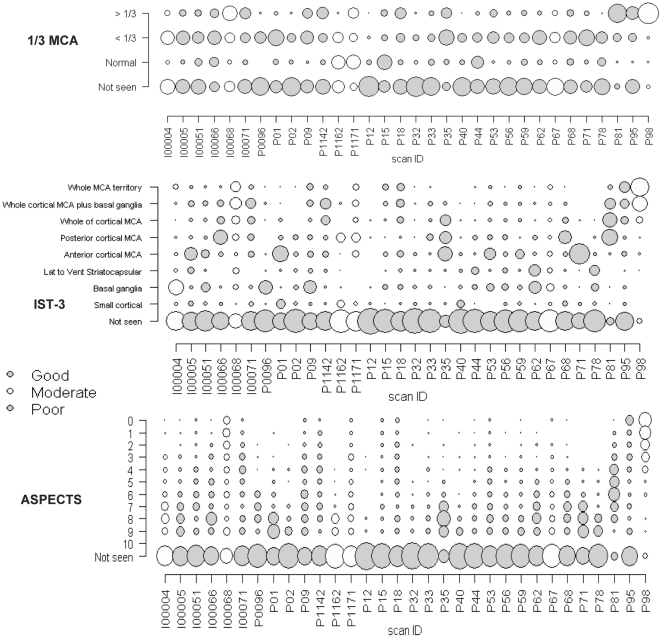
“Bubble plots” show observers' score distributions for each scan for each scoring method. The three scoring methods are the 1/3 MCA, IST-3 and ASPECTS scores. X axis indicates the individual scan identification numbers, the Y axis indicates the scores on each scale, and the scan quality as judged by the reference standard is indicated in blue (poor), moderate (yellow) or good (red).

Finally, we examined the distribution of observers' scoring of each scan on each infarct rating scale to determine equivalence of scale ratings. We plotted the frequency of scan ratings by scan and by whether the reference standard thought the scan quality was good, moderate or poor, using bubble plots (Figure 3). Here, there is one graph per rating scale, the grey shade indicates whether the scan was thought to be of good, moderate or poor quality, the size of the bubble is proportional to the number of observers giving that rating and the distribution of the bubbles on the y axis shows the spread of observers' ratings. Thus, where there are clusters of agreement, there should be larger bubbles. For all scales, there are large bubbles on the “not seen” position indicating the observers who did not identify any acute ischaemic change and therefore did not assign a rating on the scales. For the 1/3 MCA and IST-3 scores the bubbles are more clustered than for the ASPECTS score which tends to produce widely spread ratings across all possible scores. In general, a high score on the 1/3 MCA was associated with a high score on the IST-3 scale, but with less agreement on the ASPECTS score. The prominent clustering on the 1/3 MCA score may reflect the smaller number of choices available on the 1/3 MCA. The 1/3 MCA and the ASPECTS score assess the size of the ischaemic lesion but do not localise the infarct to a particular part of the MCA territory, e.g. an ASPECTS score of “7” indicates that three brain regions are abnormal but does not indicate *which* three. In contrast the IST-3 score both assesses lesion size *and* localises the lesion, hence the IST-3 score value provides greater precision of lesion location and extent.

## Discussion

We have identified factors associated with optimal detection of early ischaemic signs on CT scans in hyperacute stroke and that any of three ischaemic lesion rating scales improves lesion detection. All three scales performed similarly. Thus, physicians and radiologists involved in the care of stroke patients that are already using a rating scale should continue to use it. Those who are not may benefit from learning to use one. Acute stroke scanning departments, casualty departments and stroke assessment wards could usefully display the scales as an aide memoir for when scans are being interpreted. We showed that the main distinguishing feature between neuroradiologists (best observer performance) and other specialties was not years of experience but that neuroradiologists apparently took longer to read the scans. Although this was suggested in our previous analysis, we were not able to account for other factors which might have confounded this observation. While some patient and scan factors influenced lesion detection, reassuringly, scan quality had little effect. It is important to improve CT infarct detection as CT is more practical and accessible in hyperacute stroke pre-thrombolysis.[1], [2] Magnetic resonance (MR) is less practical with up to 45% of patients failing to complete imaging in some studies,[21] and mismatch imaging may be too non-specific for use in decision making in acute stroke.[22]

The strengths of this work are the large number of observers representing all disciplines looking after stroke and direct relevance to routine practise, the large number of scans chosen to reflect typical characteristics of acute stroke patients as well as a few chosen to show specific early ischaemic changes, and scans shown twice to determine intra-observer and training effects. The large sample size and careful choice of scans meant that we could examine the effect of reader, scan and patient characteristics on lesion detection. We directly compared all three lesion rating scales described so far for use in hyperacute stroke. We only included observers who read all scans and used exemplary statistics to account for multiple observers, multiple scans, and multiple interrelationships between scan/patient/observer features. The DBM MRMC software used to calculate the AUC was specifically designed for the situation of multiple observers and multiple scans.[19], [20] Our main outcome measures, the AUC and odds ratio, are easy to interpret. The alternative approach would be a categorical mixed model as the data from the scales are ordered categorical and a mixed model approach would be necessary to allow for the within-observer and within-scan correlations. However the parameters of a mixed model can be hard to estimate for computational reasons, they require more assumptions to be met than either the AUC or logistic regression analysis and the results are more difficult to interpret.

The study also has weaknesses. We asked observers to optimise their computer settings for scan display using the SMPTE test (and provided on line/email help) but we do not know if they used optimum viewing conditions (darkened room, etc). Modern Digital Imaging and Communications in Medicine (DICOM) image viewers allow the observer to manipulate the scan window level and width. In the version of the web viewing tool used here, the scans had been saved on optimal settings but could not be manipulated by the observers (later version of the web-based viewing tool does enable scan manipulation). We relied on the information provided by observers about who they were and their background. However, we know that their email addresses were correct and many had responded to our specific call for interested people to join the study. We do not think that anyone who was not interested in stroke would have been sufficiently motivated to spend the (approximately) 2.5 hours in total that it took to complete all 63 scans. We assumed that the observers conducted the task in a focussed way without interruption, but cannot be sure that the recorded time of scan reading was all spent in scan reading. Thus some of the apparent difference between specialties may be because some were more likely to be interrupted than others. For example, at work radiologists are interrupted on average every four minutes. However, we suspect that many of the observers read the scans in their own time and not while at work, and a different interruption rate would be unlikely to explain the difference in observer agreement or time taken to read scans between neuroradiologists and general radiologists. We were not able to include the readings of observers who only completed some of the scans, as that would have reduced the total number of scans available for analysis and hence power of the study, otherwise we could have analysed data on up to 900 observers. We did not include any scans with haemorrhagic stroke or stroke mimics, so these results to not apply to the generality of patients presenting with possible acute stroke, only to those with acute ischaemic stroke. A separate study would be required to address observer agreement for haemorrhagic stroke or distinction of stroke mimics from ischaemic or haemorrhagic stroke. The observers did not rate each scale separately from the recording of acute ischaemic change or each other. Therefore it is possible that the rating on one scale influenced the detection of acute ischaemic signs or rating on another scale. However, the scales were presented in different parts of the questionnaire and prior to the present study, had never all been tested on the same set of scans so there was little prior belief that they were all measuring the same thing. In fact one scale (IST-3) was virtually unknown, there was little evidence of use of ASPECTS except in research, leaving the 1/3 MCA as the scale that was mentioned in thrombolysis guidelines and that was likely to be in most common clinical use. While it would have been ideal to show the scans without the scales and then with each scale in turn, this would have required each observer to read the full batch of scans at least four times in total, which would have severely compromised participation.

Other studies have assessed observer reliability of CT infarct sign detection, but in general had too few readers or too few scans to provide reliable results or to look at patient/scan/observer characteristics.[9] The one previous study that did achieve a large observer group[11] had 532 observers rate 20 scans during training of investigators at an induction meeting for a multicentre trial of thrombolysis in stroke. The drawbacks were that most of the raters were from one specialty, were already experienced in stroke and it is impractical to undertake such an exercise regularly. In all 15 previous studies of inter-observer agreement for early infarct signs on CT (median 30 scans, six raters) published between 1990 and 2003, there was no information on which signs were reliably detected nor on the effect of scales, observer or patient factors.[9]

Observer reliability studies of medical image interpretation can improve patient care by providing insights into how experts perform. Such information may help improve the performance of less experienced individuals. However, observer reliability studies in general are usually too small to provide reliable estimates of agreement on specific diagnostic features or on the effect of observer/patient/image characteristics due to the simple practical constraints of providing enough scans to a large group of observers using traditional methods.[9] The use of web technology, with a quick and simple way (for the observer) to access the scans and enter their readings from anywhere with internet access and a computer at any time overcame many of these practical barriers. It is difficult to power observer reliability studies adequately. The typical response from a statistician to the question “how many cases and observers do I need” is “as many as you can get”. Using web technology, we achieved both a large number of observers and a large number of scans making the results directly translatable to routine practise. The total number of observer-scan interpretations was 11939. Other large studies of observer reliability e.g. for MR imaging of the breast,[23] have included larger numbers of scans (1541) but fewer observers (44) and each observer read variable numbers of scans in pairs (median number of scans read 37, interquartile range (IQR) 8–95.5) resulting in a total of 3082 readings – this enabled a comparison of pairs of readers but not between specialties. Our novel web based approach could be applied to other medical images (eg mammography, pulmonary CT, MR of joints) and to non-radiological applications e.g. dermatology, histology, blood smears, i.e. anything where visual interpretation of some sort of image is necessary.

What are the implications for practise? The major difference between neuroradiologists and the other specialty groups was that neuroradiologists apparently took longer to read the scans. Practice helps – this is presumably also what differentiates neuroradiologists from the rest - but not total years in training. Therefore doctors reading acute stroke CT scans should slow down, practice scan reading, look for three cardinal signs (hypoattenuation, swelling and hyperattenuated artery), be encouraged to use a scale, be aware that leukoaraiosis reduces detection of acute signs (but not old stroke lesions) and that scan quality per se does not overly reduce ischemic lesion detection. Awareness of the pathophysiology underlying each sign may help interpretation. Keeping a diagram of the preferred infarct rating scale near the reading console might help as an aide memoir. CT is likely to remain with us as the main diagnostic method for patients with hyperacute ischaemic stroke, therefore those caring for stroke patients should practice CT scan reading as much as they can. The ACCESS study is available for training at http://www.neuroimage.co.uk, where, upon completion of all 63 scans (or as many as you want to do), there is feedback on how the reader's responses compared with those who have read the scans so far, including what the reference standard, a panel of experts, and each specialty said about each scan on the first and second readings, the initial and follow-up scans are visible, and a certificate awarding up to 5 CPD credits may be downloaded.
